# Effects of Lipid-Lowering Drugs on Irisin in Human Subjects *In Vivo* and in Human Skeletal Muscle Cells *Ex Vivo*

**DOI:** 10.1371/journal.pone.0072858

**Published:** 2013-09-02

**Authors:** Ioanna Gouni-Berthold, Heiner K. Berthold, Joo Young Huh, Reena Berman, Nadine Spenrath, Wilhelm Krone, Christos S. Mantzoros

**Affiliations:** 1 University of Cologne, Center for Endocrinology, Diabetes and Preventive Medicine, Cologne, Germany; 2 Charité University Medicine Berlin, Evangelical Geriatrics Center Berlin (EGZB) and Virchow Clinic Campus, Lipid Clinic at the Interdisciplinary Metabolism Center, Berlin, Germany; 3 Section of Endocrinology, Boston VA Healthcare System and Beth Israel Deaconess Medical Center, Harvard Medical School, Boston, Massachusetts, United States of America; University of Cordoba, Spain

## Abstract

**Context and Objective:**

The myokine irisin has been proposed to regulate energy homeostasis. Little is known about its association with metabolic parameters and especially with parameters influencing pathways of lipid metabolism. In the context of a clinical trial, an exploratory *post hoc* analysis has been performed in healthy subjects to determine whether simvastatin and/or ezetimibe influence serum irisin levels. The direct effects of simvastatin on irisin were also examined in primary human skeletal muscle cells (HSKMCs).

**Design and Participants:**

A randomized, parallel 3-group study was performed in 72 men with mild hypercholesterolemia and without apparent cardiovascular disease. Each group of 24 subjects received a 14-day treatment with either simvastatin 40 mg, ezetimibe 10 mg, or their combination.

**Results:**

Baseline irisin concentrations were not significantly correlated with age, BMI, estimated GFR, thyroid parameters, glucose, insulin, lipoproteins, non-cholesterol sterols, adipokines, inflammation markers and various molecular markers of cholesterol metabolism. Circulating irisin increased significantly in simvastatin-treated but not in ezetimibe-treated subjects. The changes were independent of changes in LDL-cholesterol and were not correlated with changes in creatine kinase levels. In HSKMCs, simvastatin significantly increased irisin secretion as well as mRNA expression of its parent peptide hormone FNDC5. Simvastatin significantly induced cellular reactive oxygen species levels along with expression of pro- and anti-oxidative genes such as Nox2, and MnSOD and catalase, respectively. Markers of cellular stress such as atrogin-1 mRNA and Bax protein expression were also induced by simvastatin. Decreased cell viability and increased irisin secretion by simvastatin was reversed by antioxidant mito-TEMPO, implying in part that irisin is secreted as a result of increased mitochondrial oxidative stress and subsequent myocyte damage.

**Conclusions:**

Simvastatin increases irisin concentrations *in vivo* and *in vitro*. It remains to be determined whether this increase is a result of muscle damage or a protective mechanism against simvastatin-induced cellular stress.

**Trial Registration:**

ClinicalTrials.gov NCT00317993 NCT00317993.

## Introduction

Irisin is a newly identified hormone secreted by myocytes (i.e., a myokine) that has been proposed to mediate beneficial effects of exercise and to influence multiple metabolic pathways, such as energy and glucose metabolism [1]–[3]. Regulated by PPARγ coactivator-1alpha (PGC-1α), irisin is proteolytically processed from the product of the fibronectin type-III domain containing protein 5 (FNDC5) gene prior to being released into the circulation. Irisin induces browning of subcutaneous adipocytes and thermogenesis by increasing uncoupling protein-1 (UCP1) levels in both cell cultures and mouse models [3]. Considering the metabolic benefits of “beige/brite (brown-in-white) fat” formation [4], [5], irisin administration has been proposed as a potential therapeutic tool to treat obesity and diabetes [6] and thus may have implications for decreasing cardiovascular risk.

A previous study from our group has suggested that age and muscle mass are the primary predictors of circulating irisin concentrations, with young male athletes having several-fold higher levels than middle-aged obese women [7]. Furthermore, irisin levels increased in response to acute exercise and decreased after surgically induced weight loss in parallel to a decrease in body mass [7]. Still, there is little knowledge regarding the way irisin levels are regulated, for example by parameters involved in lipoprotein metabolism pathways. Statins are widely used in the treatment of hypercholesterolemia and for reducing cardiovascular risk [8]. This becomes of particular interest given recent data indicating that statin treatment and increased cardiovascular fitness, which would be expected to elevate irisin levels, are both associated with low mortality among dyslipidemic individuals [9]. The combination of statin treatment and increased fitness resulted in substantially lower mortality risk than either alone, reinforcing the importance of physical activity for individuals with dyslipidemia. Whether, similar to physical activity, statins may also increase irisin levels and thus affect metabolism and improve risk among dyslipidemic individuals through the irisin pathway too remains to be explored.

The current clinical trial is to our knowledge the first study to examine whether lipid-lowering drugs affect circulating irisin levels. In this study we aimed to determine the effects of simvastatin and ezetimibe on irisin by conducting a randomized study in humans, and to characterize the parameters that predict baseline irisin levels in this cohort *in vivo*.

To find the mechanisms related at the molecular level, we also studied the effects of simvastatin on cellular stress markers as well as on FNDC5/irisin expression and secretion in primary human skeletal muscle cells (HSKMCs).

## Subjects and Methods

### Study design

The study design has been published before [10]–[14]. In short, the present clinical trial was a single-center prospective, randomized, parallel 3-group open-label study (N = 24 subjects for each group). The participants were randomized to receive simvastatin (40 mg/day), ezetimibe (10 mg/day), or simvastatin (40 mg/day) plus ezetimibe (10 mg/day) for a period of 2 weeks. Blood was drawn in the morning after a 12-h fast at days 1 (before the initiation of treatment) and 15 (at the end of the 2-week treatment period). The original study has been registered at ClinicalTrials.gov NCT00317993.

### Subjects

The study protocol of the clinical trial was approved by the Ethics Committee of the University of Cologne, and all subjects gave written informed consent. In the *ex vivo* study, each subject had given written informed consent before taking part in the study as approved by the Institutional Review Board at Beth Israel Deaconess Medical Center. For the clinical trial, seventy-two male volunteers were recruited by word of mouth and through advertisements in the Cologne area and on campus. Inclusion criteria were age between 18 and 60 years, body mass index (BMI) between 18.5 and 30 kg/m^2^, LDL cholesterol concentrations <190 mg/dl, triglycerides <250 mg/dl and normal blood pressure (<140/90 mmHg). Individuals who had received lipid-lowering drugs within 12 weeks prior to study entry, those with a history of excessive alcohol intake, liver disease, renal dysfunction (estimated glomerular filtration rate <60 ml/min), rheumatologic disease, coronary heart disease, diabetes or other endocrine disorders, eating disorders, history of recent substantial (>10%) weight change, history of obesity (body mass index >35 kg/m^2^) or taking medications known to affect lipoprotein metabolism or the immune system were excluded from the study. All patients were advised to keep their usual dietary habits and their usual exercise levels throughout the trial.

### Biochemical assays

Lipoproteins were analyzed on the day of blood collection in the core laboratory of the Cologne University Medical Center. Irisin was measured using a commercially available ELISA kit as previously reported (sensitivity, 8.3 ng/ml; intra-assay coefficient of variation, 4–6%) [7]. Creatine kinase was measured using an automated analyzer (Hitachi cobas c311; Roche Diagnostics, Indianapolis, IN). Insulin levels were measured by RIA (Diagnostic System Laboratories, Webster, TX; sensitivity, 1.3 µU/ml; inter- and intra-assay CVs, 4.7–12.2% and 4.5–8.3%, respectively). Insulin resistance was estimated at baseline using the homeostasis model assessment (HOMA) index―the product of fasting glucose (mmol/l) and insulin (µU/ml) divided by the constant 22.5. Serum high-sensitivity C-reactive protein was determined using the Quantikine Human C-reactive protein immunoassay (R&D Systems, Minneapolis, MN). Interleukin-6 was determined using the human IL-6 Platinum ELISA (eBioscience Diagnostics, San Diego, CA). Serum adiponectin, leptin, and resistin levels were measured using radioimmunoassays (Linco Research, St. Charles, MO, and BioVendor, Brno, Czech Republic), as previously described [15]–[17], and high-molecular weight (HMW) adiponectin was measured using an ELISA (ALPCO Diagnostics, Salem, NH) as previously described [18]. The sensitivity of the adiponectin assay was 1 ng/ml, intra-assay CV of 6.6%; resistin 0.2 ng/ml, CV 3.4–5.2%; leptin 0.5 ng/ml, CV 6–7%; and HMW adiponectin 0.04 ng/ml and CV 2.8–8.4%. Coenzyme Q10 was analyzed using HPLC as previously described [14]. Plasma concentrations of Lp-PLA2 were measured using ELISA (USCN Life Science Inc., Wuhan, China). All other parameters were measured using standard laboratory methods in the core laboratory.

### Primary human skeletal muscle cell culture

Abdominal/thigh muscle tissue was collected from patients undergoing surgery [7]. Each subject had given written informed consent before taking part in the study as approved by the Institutional Review Board at Beth Israel Deaconess Medical Center. Biopsied skeletal muscle tissue was immediately placed into dissociation media containing 0.1% BSA, 0.25% trypsin-EDTA, and 0.1% collagenase. Then the tissue was minced into small pieces and was incubated in 37°C water bath for 1 hr. After centrifugation, the pellet was resuspended in Skeletal Muscle Cell Growth Media (PromoCell, Heidelberg, Germany) and plated on T25 culture dish. After reaching 70–80% confluence, growth media was switched to Skeletal Muscle Cell Differentiation Media (PromoCell) for the differentiation of myoblasts into myotubes. After 5 days of differentiation, the media was changed back to growth media for additional 2–4 days for a stable differentiation, according to the manufacturer's instructions. A therapeutic dose of 40–80 mg/day simvastatin *in vivo* corresponds to approximately 5 µM simvastatin *in vitro* [19]–[21] and therefore, doses ranging from 1 to 10 µM simvastatin were used in this study. For antioxidant experiments, 100 µM mito-TEMPO (Santa Cruz Biotechnology, Dallas, TX) was administered 1 hr before simvastatin treatment.

### Gene expression analysis

Total RNA was extracted from HSKMCs using Trizol (Invitrogen, Carlsbad, CA) according to a standard protocol. mRNA levels of FNDC5, PGC-1α, atrogin-1, Nox2, Nox4, MnSOD, and catalase were measured using TaqMan Gene Expression Assays (Assay id: Hs00401006_m1, Hs01016719_m1, Hs01041408_m1, Hs00166163_m1, Hs00418356_m1, Hs00167309_m1, Hs00156308_m1, respectively). Briefly, TaqMan Gene Expression Assay primer mix and TaqMan PCR Master Mix (Applied Biosystems, Foster City, CA) were added to wells containing equal amounts of cDNA from HSKMCs. mRNA expression was measured by real-time PCR using the comparative CT method (ABI7500 FAST, Applied Biosystems). Data were normalized to β-actin (id: Hs99999903_m1) in each reaction.

### Irisin secretion in cell lysates and media

Cell lysates and the culture media were collected 24 and 48 hrs after simvastatin treatment. After centrifugation to remove cell debris, irisin was measured in cell lysates and culture media using a commercially available kit as described above.

### Morphological analysis

For morphological image analysis, the cells were photographed using a light microscope (Carl Zeiss, Thornwood, NY). To quantify the effect of simvastatin on myotube thinning, the diameter of myotubes were measured as previously described [22]. Briefly, 4 random fields were selected to measure the muscle fiber size using Image J software (Scion, Frederick, MD).

### Measurement of oxidative stress

Differentiated HSKMCs were incubated with 2 and 10 µM simvastatin for 48 hrs and then loaded with the peroxide-sensitive fluorophore 2′,7′-dichlorofluorescein diacetate (DCF-DA; 5 µM; Molecular Probes, Carlsbad, CA) for 20 min. Then, the fluorescent intensity was measured using a fluorimeter (BioTek, Winooski, VT) at 485 nm excitation and 530 nm emission. The fluorescence was measured in triplicate to avoid well-to-well variation and was normalized by protein concentration.

### Western blot analysis

After cells have been treated with 5 µM simvastatin for 24 and 48 hrs proteins were extracted with lysis buffer containing 20 mmol/l Tris-HCl (pH 7.4), 150 mmol/l NaCl, 5 mmol/l EDTA, 0.1 mmol/l phenylmethylsulfonyl fluoride, 0.05% aprotinin, and 0.1% Igepal and then incubated for 30 min at 4°C. The suspension was centrifuged for 15 min at 13,000 rpm, and the supernatant was saved as the protein extract. After SDS-PAGE, proteins were transferred to nitrocellulose membranes. The membranes were blocked for 1 hr in Tris-buffered saline (TBS) containing 5% nonfat dry milk and 0.1% Tween 20 and then incubated with Bcl-2 and Bax antibody overnight (Santa Cruz Biotechnology). Measurement of signal intensity was performed using Image J processing and analysis software.

### Cell viability assay

Cell viability was measured using the 3-(4,5-dimethylthiazol-2-yl)-2,5-diphenyl tetrazolium bromide (MTT) assay kit (Sigma-Aldrich, St. Louis, MO), according to the manufacturer's instructions.

### Data analysis

Statistical analyses were performed using Stata11 (StataCorp LP, College Station, TX). Descriptive statistics are presented as counts (percentage proportions) or means ± SD. The main outcome parameters in the first part of the clinical trial were Pearson's or Spearman's rank correlation coefficients (as appropriate) to estimate associations between baseline values of irisin and other baseline parameters. Stepwise linear regression analyses (bidirectional elimination procedure) were also performed to identify parameters for model building. The main outcome parameter in the second part of the clinical trial was percent change in irisin concentrations from baseline after 2 weeks treatment with lipid-lowering drugs. We used Student's paired t-test for statistical comparisons within the groups. All tests were performed two-sided. P-values are presented descriptively. HSKMC data are presented as means ± SEM. Mean values obtained from each group were compared using ANOVA with subsequent Fisher's significant difference method.

## Results

### Cross-sectional clinical trial: Correlation of irisin with baseline parameters

**Figure 1:**
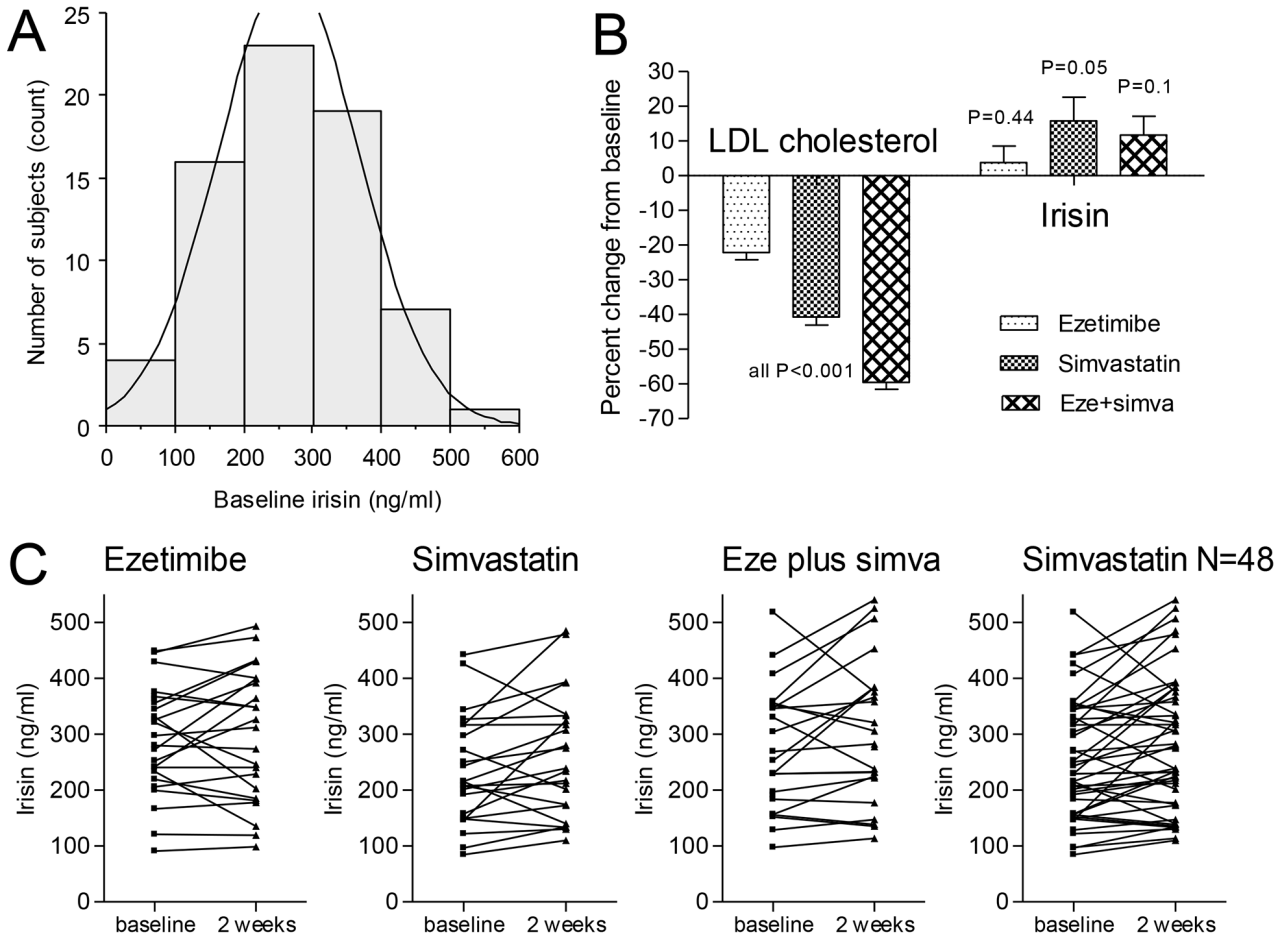
Irisin and LDL cholesterol concentrations at baseline and after 2 weeks of treatment. (A) Frequency histogram and normal distribution of irisin baseline concentrations. Data of N = 70 subjects were available. Mean ± SD baseline concentrations were 265±102 ng/ml (range 85 to 518 ng/ml). (B) Percent change from baseline concentrations in LDL cholesterol (left) and irisin (right). (C) Individual changes from baseline to 2-weeks irisin concentrations in the 3 treatment groups. The graph on the right shows the combined data of the subjects receiving simvastatin (either alone or in combination with ezetimibe).

The baseline subject characteristics are shown in Table 1. The mean age of the subjects was 31.5±9.2 yrs (range 20–60 yrs), mean body weight 85±12 kg (range 64–115 kg), and mean BMI was 25.7±3.2 kg/m^2^ (range 19.5–32.8 kg/m^2^). The baseline subject characteristics were not different between the groups. Circulating irisin concentrations were measured in 70 out of the 72 subjects (due to missing samples in 2) and had a mean value of 265±102 ng/ml, ranging from 85–518 ng/ml (Figure 1A).

**Table 1:** Baseline characteristics of the randomized patients and correlation analyses between baseline values and baseline irisin plasma concentrations.*

Parameter	Baseline values	Correlation coefficient**	P value
**Demographic characteristics**			
Age (years)	31.5±9.2	–0.14	0.26
Smoking status (N and %)			
Current smokers	21 (29%)		
Former smokers	9 (13%)		0.23§
Never smokers	42 (58%)		
**Anthropometric characteristics, body composition and clinical parameters**			
Height (cm)	181±7	–0.03	0.78
Weight (kg)	85±12	–0.14	0.25
Body mass index (kg/m^2^)	25.7±3.2	–0.14	0.26
BIA body fat (%)	21.4±5.7	–0.03	0.82
BIA lean body mass (%)	66.0±6.4	–0.18	0.14
BIA ratio ECM/BCM	0.82±0.09	0.07	0.58
Creatinine (mg/dl)	0.96±0.12	0.06	0.62
Estimated glomerular filtration rate (ml/min)	135±25	–0.01	0.94
**Hormonal parameters**			
Thyroid stimulating hormone (mU/l)	1.65±0.82	–0.10	0.42
Free triiodothyronine (ng/l)	3.7±0.4	–0.005	0.97
Free thyroxine (ng/l)	13.7±1.7	0.10	0.40
**Glucose and lipid metabolism**			
Fasting blood glucose (mg/dl)	88±8	0.03	0.84
Fasting insulin (mU/l)	9.9±8.4	0.12	0.32
HOMA index	2.2±1.8	0.19	0.13
**Lipid metabolism parameters**			
Total cholesterol (mg/dl)	189±35	–0.18	0.14
LDL cholesterol (mg/dl)	111±30	–0.11	0.37
Non-HDL cholesterol (mg/dl)	126±33	–0.12	0.32
HDL cholesterol (mg/dl)	64±15	–0.16	0.20
Triglycerides (mg/dl)	95±43	–0.11	0.38
Lipoprotein(a) (mg(dl)	16±24	–0.27	0.02
**Non-cholesterol sterols**			
Campesterol¶	208±83	0.04	0.72
Lathosterol¶	138±40	–0.05	0.69
Ratio campesterol/lathosterol (mg/mg)	1.8±1.3	–0.01	0.97
**Adipokines/myokines**			
Resistin (ng/ml)	14.1±5.3	0.04	0.77
Adiponectin (µg/ml)	13.4±5.3	0.10	0.43
High molecular weight adiponectin (µg/ml)	2.7±2.0	0.13	0.28
Leptin (µg/ml)	3.0±2.8	0.02	0.85
Irisin (ng/ml)	265±102	1.00	
**Inflammation markers**			
High-sensitivity C-reactive protein (mg/l)	0.75±0.93	–0.12	0.34
Interleukin-6 (ng/l)	0.94±1.26	–0.19	0.12
Lipoprotein-associated phospholipase A_2_ (ng/ml)	141±194	–0.09	0.48
Blood neutrophil count (10^3^/µl)	2.8±1.0	–0.10	0.40
**Molecular markers of cholesterol metabolism**			
LDL receptor protein (PBMC)†	17.6±9.3	0.01	0.93
HMG-CoA reductase mRNA‡	3.3±1.3	–0.15	0.21
LDL receptor mRNA‡	0.52±0.32	–0.40	0.004
NPC1L1 mRNA‡	33±31	–0.29	0.02
PCSK9 mRNA‡	1.7±0.1	–0.02	0.87
PCSK9 (ng/ml)	52±20	0.01	0.94
Coenzyme Q10 (µg/ml)	0.99±0.3	–0.22	0.06

* Continuous variables are expressed as means ± SD (for smoking status N and percentage of total) of N = 72 patients.

** Pearson's or Spearman's rank correlation coefficient, where appropriate.

§ Analysis of variance.

¶ The data indicate the ratio of the respective non-cholesterol sterol to cholesterol (µg/mg) ×100.

† LDL receptor protein is given as flow cytometry-specific fluorescence, calculated by subtracting the nonspecific fluorescence intensity from the total fluorescence intensity.

‡ Gene expression is given as number of the respective mRNA copies divided by the number of copies of the TATA housekeeping gene mRNA.

BIA, bioelectrical impedance analysis.

HOMA, homeostasis model assessment.

We performed bivariate regression analysis of irisin concentrations and the demographic, anthropometric, hormonal, and metabolic parameters in order to identify parameters that may influence irisin baseline concentrations (Table 1). The parameters that were found to be significantly correlated with irisin were lipoprotein(a) (R = –0.27), coenzyme Q10 (R = –0.22), and the mRNA levels of LDL receptor (R = –0.40) and NPC1L1 (R = –0.29).

None of the other parameters was significantly correlated with irisin. However, there were weak negative correlations with age, BMI, lean body mass, all lipoprotein fractions, and inflammation markers (hsCRP, IL-6, Lp-PLA2 and blood neutrophil count). There were no relevant correlations with smoking status, kidney or thyroid function parameters, non-cholesterol sterols, and adipokines (resistin, adiponectin, and leptin).

Stepwise linear regression analysis identified only 2 parameters to have a significant correlation with irisin concentrations, namely total cholesterol (beta coefficient –0.79, SE 0.37, F-to-remove 4.6) and lipoprotein(a) (beta coefficient –2.68, SE 0.82, F-to-remove 10.7). The model had an adjusted R squared of 0.24, F = 8.5, P = 0.0007.

### Interventional clinical trial: Effect of lipid-lowering drugs on irisin levels

The effects of the lipid-lowering drugs on lipoprotein concentrations were of expected magnitude and were reported before in detail [12]. In short, ezetimibe decreased the main outcome parameter low-density lipoprotein (LDL) cholesterol by 22±10%, simvastatin by 41±12%, and the combination of the two drugs by 60±10% (all P<0.001; Figure 1B). The changes of circulating irisin in the three treatment groups were +3.8±22.7% (P = 0.44), +15.8±32.5% (P = 0.05), and +11.7±25.3% (P = 0.1), respectively. When combining the two groups that received simvastatin (N = 48), the mean percent change of irisin was +13.8±28.9% (P = 0.01) with a mean increase from baseline of +28 ng/ml (95% CI +7 to +49 ng/ml). The mean difference in the ezetimibe group, however, was only +10 ng/ml (95% CI –17 to +38 ng/ml). Figure 1C shows the individual irisin data in the three groups and in the combined simvastatin group. There was no correlation between changes in irisin concentrations and changes in LDL cholesterol concentrations. However, there was a highly significant intra-subject correlation between pre- and post-treatment irisin concentrations (R = 0.82, P<0.0001). There was no correlation between the change in irisin levels by simvastatin treatment and the respective changes in lipoprotein(a), coenzyme Q10, and mRNA levels of LDL receptor and NPC1L1 (Niemann-Pick C1-like protein 1).

**Figure 2:**
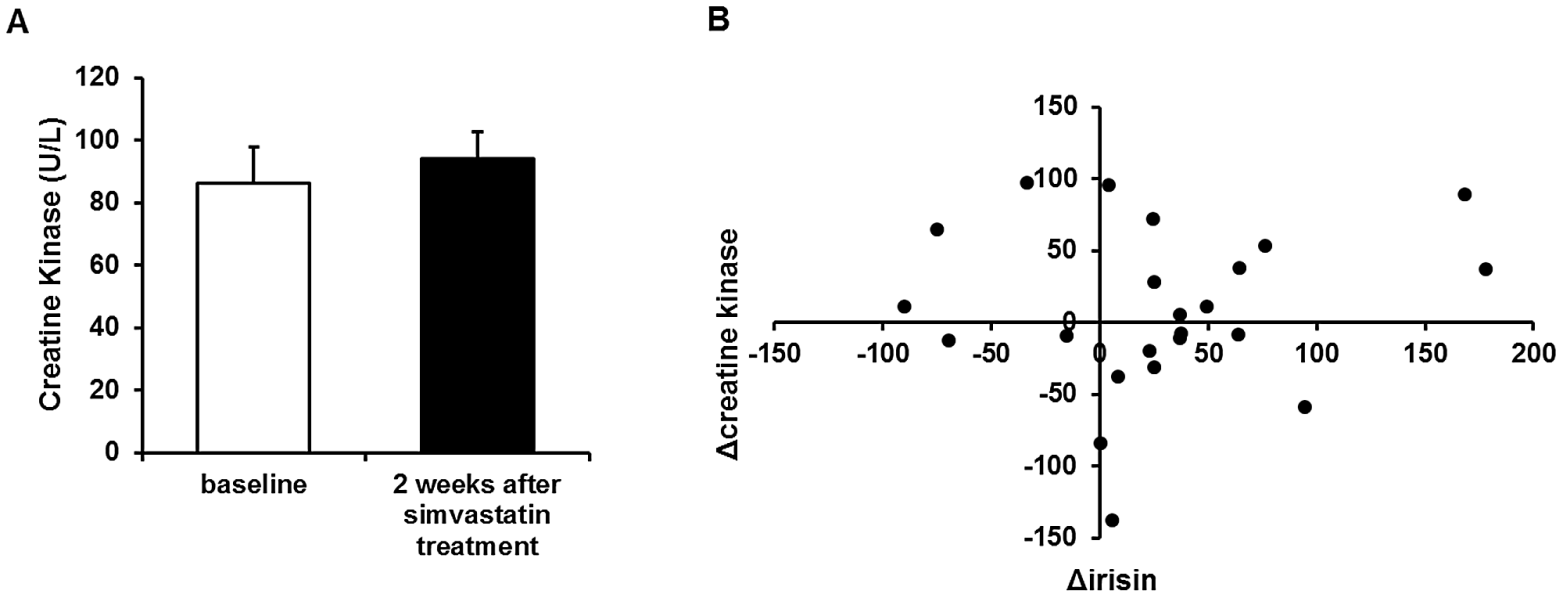
Increased irisin does not correlate with circulating creatine kinase levels. (A) Serum creatine kinase levels before and 2 weeks after simvastatin treatment. (B) Correlation between change in irisin levels and change in creatine kinase levels in simvastatin-treated subjects. Values are means ± SEM of N = 23 subjects.

Since simvastatin has been known to induce muscle damage, we measured creatine kinase levels and their correlation with circulating irisin. As shown in Figure 2A and 2B, serum creatine kinase levels were not different before and after simvastatin treatment (P = 0.54), and changes in creatine kinase levels did not correlate with changes in irisin levels in these patients (R = 0.0123). This suggests that the increase in irisin levels in the simvastatin-treated group was not due to considerable muscle damage.

### In vitro studies

#### Effects of simvastatin on FNDC5/irisin expression and secretion in primary HSKMCs

**Figure 3:**
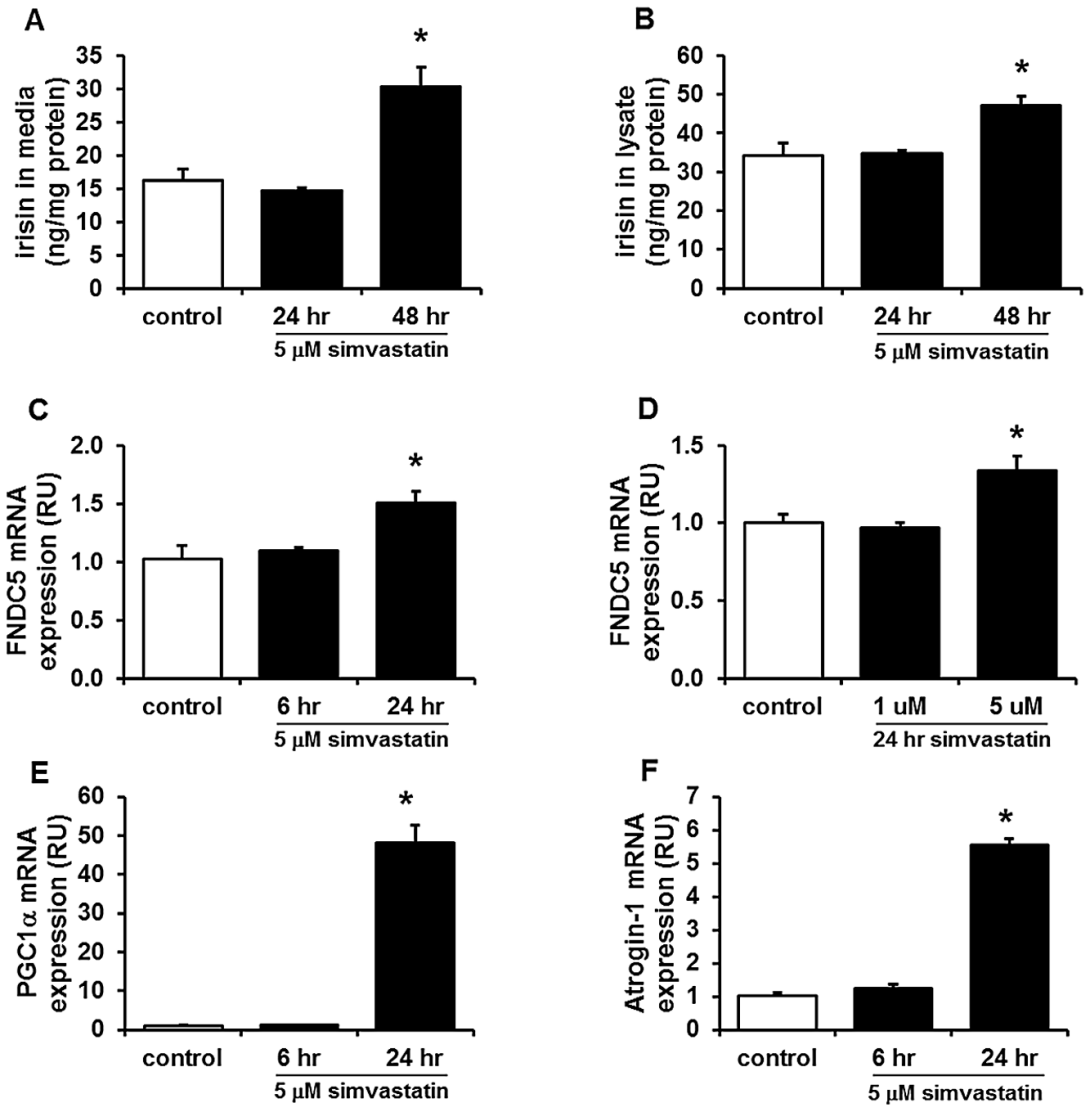
Simvastatin increases irisin secretion and FNDC5 mRNA expression in human skeletal muscle cells (HSKMCs). Measurement of irisin in media (A) and lysate (B) at 24 and 48 hrs after 5 µM simvastatin treatment. FNDC5 mRNA levels were measured by real-time PCR after 5 µM simvastatin treatment for 6 and 24 hrs (C) or 1 and 5 µM for 24 hrs (D). mRNA levels of PGC-1α (E) and atrogin-1 (F) were also measured in simvastatin-treated HSKMCs (5 µM, 6 and 24 hrs). Values are means ± SEM of 4 individual experiments. *P<0.05 *vs.* control.

To assess the effect of simvastatin on irisin more directly, we used primary HSKMCs isolated from human muscle biopsies. Measurement of irisin in media after 5 µM simvastatin treatment resulted in significantly increased secretion of irisin compared to control after 48 hrs (Figure 3A). Interestingly, irisin levels were also increased in the HSKMC lysates of simvastatin-treated cells (Figure 3B). As shown in Figure 3C and 3D, FNDC5 mRNA levels were upregulated 24 hrs after 5 µM simvastatin treatment. mRNA levels of PGC-1α, a known upstream regulator of FNDC5 [3], and atrogin-1, a known marker of muscle atrophy development [23], [24], were also significantly upregulated 24 hrs after 5 µM simvastatin treatment (Figure 3E and 3F).

#### Effects of simvastatin on oxidative stress and cell viability in primary HSKMCs

**Figure 4:**
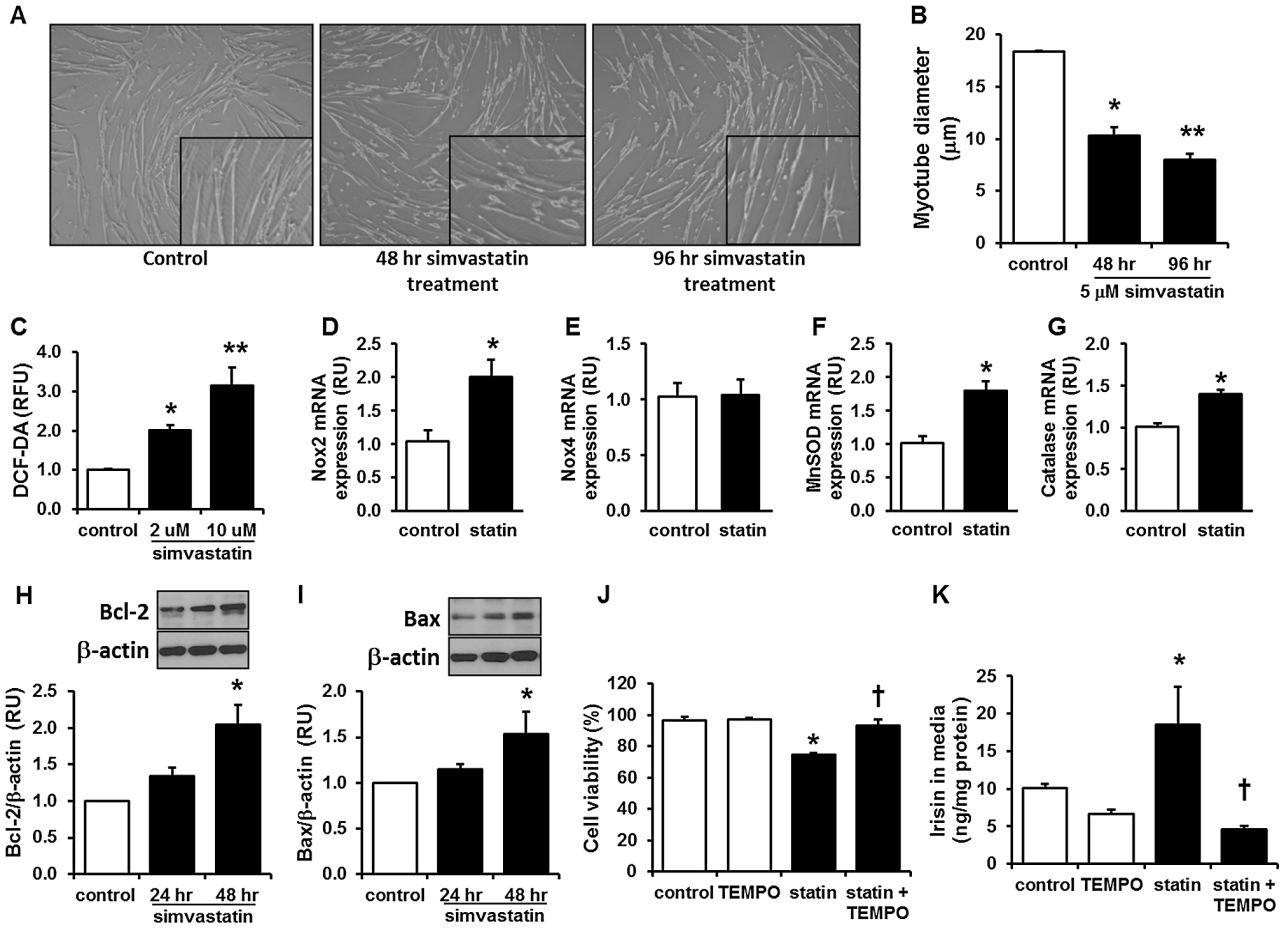
Simvastatin induces muscle damage, oxidative stress, and apoptosis in human skeletal muscle cells, and mito-TEMPO reverses simvastatin-induced cellular damage and irisin secretion. (A) Differentiated HSKMCs were incubated with 5 µM simvastatin for 48 and 96 hrs and representative pictures were taken. Enlarged inset pictures are shown for better viewing. (B) The myotube diameter was measured in the images shown in (A) using Image J, as described in the methods. (C) Intracellular oxidative stress levels were measured with DCF-DA 48 hrs after 2 and 10 µM simvastatin treatment. (D–G) Expression of oxidative stress related genes, including Nox2, Nox4, MnSOD, and catalase were measured 24 hrs after 5 µM simvastatin treatment. (H–I) Protein levels of Bcl-2 and Bax after 24 and 48 hrs 5 µM simvastatin treatment and its quantification in HSKMCs. (J) Cell viability was measured by MTT assay after 5 µM simvastatin treatment for 48 hrs. (K) Irisin secretion in media was measured 48 hrs after 5 µM simvastatin treatment. For (J) and (K), 100 µM Mito-TEMPO was administered for 1 hr prior to simvastatin treatment. Values are means ± SEM of 4 individual experiments. RFU: relative fluorescence unit, Statin: simvastatin, TEMPO: mito-TEMPO. *P<0.05 *vs.* control, **P<0.05 *vs.*48 hr treated (B) or 2 µM simvastatin-treated (C) HSKMCs, †P<0.05 *vs.* simvastatin-treated HSKMCs.

As shown in Figure 4A, 48 and 96 hrs of 5 µM simvastatin induced fiber thinning in HSKMCs with significantly reduced myotube diameter (Figure 4B). The change in morphology was correlated with increased intracellular oxidative stress (Figure 4C) and induction by 2 µM and 10 µM simvastatin of genes regulating reactive oxygen species such as Nox2, MnSOD, and catalase whereas Nox4 remained unaltered (Figures 4D–4G). Simvastatin treatment (5 µM) for 48 hrs also significantly increased anti-apoptotic Bcl-2 and pro-apoptotic Bax protein expression (Figure 4H and 4I) and subsequently reduced cell viability by 20% (Figure 4J).

#### Effects of mitochondrial antioxidant on simvastatin-induced changes in primary HSKMCs

To find a link between simvastatin-induced oxidative stress and irisin in HSKMCs, mitochondria-targeted antioxidant mito-TEMPO was used. As shown in Figure 4J, reduction in cell viability induced by 48 hrs of simvastatin treatment was reversed by mito-TEMPO pretreatment. Moreover, mito-TEMPO treatment was sufficient to block the irisin secretion induced by simvastatin (Figure 4K), implying that irisin may be induced by simvastatin through an oxidative stress-mediated mechanism in HSKMCs.

## Discussion

This is the first prospective clinical trial investigating effects of lipid-lowering agents on irisin concentrations in humans and the first molecular study on the effect of simvastatin on irisin in human myocytes. The main finding of our study is that circulating irisin is increased in simvastatin-, but not in ezetimibe-, treated subjects independently of its lipid-lowering effect. In addition, this increase was neither correlated with creatine kinase levels nor with any clinical symptoms of myopathy. Our results from experiments in primary human muscle cells showed that simvastatin directly upregulated PGC-1α and FNDC5 expression and increased irisin secretion *in vitro*, partly through simvastatin-induced oxidative stress.

The fact that circulating irisin was increased by simvastatin and not by ezetimibe, a selective cholesterol absorption inhibitor [25], [26], could be interpreted as suggesting that the increase in irisin secretion may be related to muscle damage [27]. Simvastatin has been reported to upregulate PGC-1α and to decrease oxidative stress and apoptosis in cardiac muscle [28]. However, this upregulation is not observed in skeletal muscle from subjects with statin-induced myopathy [28]. Although the therapeutic effects of statins for managing cholesterol levels are well established, some statins (particularly lipophilic statins) are known to induce myopathy or rhabdomyolysis by mechanisms not yet fully understood. Though muscle weakness and pain are clinically much more frequent than rhabdomyolysis, it is hard to quantitate the frequency of these symptoms, since markers of muscle damage such as increased creatine kinase levels are often absent in these patients [29], [30]. We found no correlation between irisin and creatine kinase levels, a currently accepted marker of muscle damage, indicating either that the increase in irisin is not related to muscle damage or that irisin is a marker of damage much more sensitive than creatine kinase. Of note, we report a borderline significant negative correlation between baseline coenzyme Q10 levels and baseline irisin (R = –0.22, P = 0.06). Statin therapy has been associated with decreased Q10 levels and it has been suggested that low Q10 levels may promote statin-associated myopathies as a result of mitochondrial damage [14]. Whether the increase in irisin is a specific response to simvastatin treatment, as indicated by our *in vitro* data, or the unspecific result of muscular cell damage or other, unknown mechanisms, remains to be confirmed by future studies.

In contrast to the *in vivo* data, our *in vitro* data showed that simvastatin, in a dose corresponding to the therapeutic dose *in vivo*, significantly increased atrogin-1 mRNA expression, cellular reactive oxygen species levels, and markers of apoptosis, and there was a parallel induction of FNDC5 mRNA and irisin secretion in HSKMCs. Galtier *et al.* have reported that in healthy volunteers who received a high dose of simvastatin (80 mg/d), subjects with the highest creatine kinase increase displayed alterations in mitochondrial respiration and muscle calcium homeostasis, as reflected by a significantly lower Vmax rotenone succinate and an increase in Ca^2+^ spark amplitude, respectively [31]. Also, several studies have shown that simvastatin-induced mitochondrial dysfunction leads to increased oxidative stress resulting in induction of PGC-1α, which is an important regulator of mitochondrial biogenesis and metabolism [28]. Moreover, it has been recently shown that simvastatin attenuates increases in cardiorespiratory fitness and skeletal muscle mitochondrial content (citrate synthase enzyme activity) when combined with exercise training in overweight or obese patients at risk of the metabolic syndrome [32].

In line with these previous reports, genes related to mitochondrial oxidative stress regulation were elevated in our study, including MnSOD, by simvastatin treatment. Along with elevated oxidative stress, pro-apoptotic Bax protein expression was increased by simvastatin, with subsequent reduction in cell viability. Expression of anti-apoptotic protein Bcl-2 was also increased, suggesting a protective role against simvastatin-induced cellular damage, similar to previous observations [19]. Importantly, mitochondria-targeted antioxidant mito-TEMPO treatment was able to reverse the reduced cell viability as well as increased irisin secretion by simvastatin in HSKMCs, implying that irisin secretion by simvastatin could be related to oxidative stress. To date, PGC-1α is the only known inducer of FNDC5 and indeed our data show that simvastatin treatment induced both PGC-1α and FNDC5 expression in HSKMCs. Interestingly, irisin levels were increased not only in the media but also in cell lysates by simvastatin. This may either be a result of increased synthesis through observed induction of FNDC5 transcription or an indication of increased storage of this protein, which has yet to be elucidated. Moreover, whether the increase in irisin/FNDC5 mediates a “mitohormesis” mechanism downstream of PGC-1α or is simply a result of cellular damage remains to be investigated.

Statins exert beneficial effects in both primary and secondary cardiovascular disease prevention based on their potent cholesterol-lowering effects [33]. However, the overall benefits observed appear to occur much earlier and seem to be greater than what might be expected from changes in lipid levels, suggesting effects beyond cholesterol lowering alone. Various of such “pleiotropic effects” of statins have been implicated in their antiatherosclerotic effects [34]. Regarding the proposed beneficial effect of irisin on ‘browning’ of subcutaneous white adipose tissue and whole body metabolism [35], it could be postulated that an increase in irisin by statins could be beneficial, for example by influencing adipose tissue metabolism and insulin resistance. Although it has been suggested that statins may decrease body weight in individuals with type 2 diabetes [36], which would be in line with irisin-mediated weight reducing effects [7], no significant reports on weight change have been reported in the majority of statin trials [37]. Of note, the long-term diabetogenic effects of statins may be mediated via the mitochondrial damage they induce since the latter has been associated with insulin resistance [38]. In this context, considering that irisin has been found to be low in patients with type 2 diabetes [39], the statin-associated increase in irisin concentrations may indeed represent a protective compensatory reaction of the muscle cell.

The present study investigated cross-sectionally a wide variety of parameters that may predict baseline irisin concentrations in healthy men. The results of our previous study [7] suggested that there are positive correlations of irisin with muscle mass, BMI, glucose, ghrelin, and IGF-1 and negative correlations with age, insulin, cholesterol and adiponectin levels. Recently, Moreno-Navarrete *et al.* found an association between FNDC5 expression in muscle and BMI in obese subjects [40]. However, here we found no significant correlations of circulating irisin levels with the majority of the parameters studied. This may be due to the different study sample characteristics (women *vs.* men, obese *vs.* lean, older *vs.* younger) or may be attributed to a smaller number of subjects, the clinical characteristics of whom showed limited variation in the present study. This may have reduced the capacity to demonstrate significant associations. Nevertheless, one of the strongest (negative) correlations was the one with NPC1L1 expression. NPC1L1 is critical for the uptake of cholesterol across plasma membrane of the intestinal enterocyte, and is the direct target of ezetimibe [41]. Recently, it has been shown that NPC1L1 has a surprising number of new functions, beyond mediating cholesterol transport in the small intestine [42]. In specific, NPC1L1 seems to be involved in energy homeostasis, as Labonte *et al.* recently showed that NPC1L1 knock-out mice are resistant to diet-induced obesity and hyperglycemia [43]. Moreover, we found a negative correlation between irisin and LDLR expression. It has been recently shown that LDLR-related protein 6 (LRP6), a member of the LDLR family, regulates body fat and glucose homeostasis by modulating PGC-1α expression [44]. PGC-1α is also known to downregulate LDLR [45] and stimulate SREBP2-mediated transactivation of human NPC1L1 [46]. Collectively, the close relationship between NPC1L1, LDLR and irisin in our study suggests that irisin may play a role in cholesterol homeostasis or *vice versa*.

Recently, it has been shown that long-term statin treatment is associated with the development of type 2 diabetes through unknown mechanisms [47], [48]. Given the improvement in obesity and glucose homeostasis in FNDC5 overexpressed mice [7] and the inverse association between irisin concentrations and prevalent type 2 diabetes [49], increased irisin levels by statin treatment (as observed in our study) may simply reflect a compensatory mechanism activated to limit other, deleterious effects of statins. We have previously reported that circulating irisin levels are positively correlated with glucose and negatively correlated with age, insulin, cholesterol, and adiponectin levels, indicating a feedback loop of irisin in response to deterioration of insulin sensitivity and glucose/lipid metabolism [7].

Strengths of our study are its novelty in terms of studying a new metabolically important hormone, its randomized design and robust statistical methodology, the blinded measurements of all end-point parameters, the excellent treatment adherence (99%, as determined by pill count) and the use of a “drug-naïve” population, devoid of co-medications or co-morbidities which could potentially alter the results. Limitation of our study includes the short treatment period which could not have been sufficient to detect the full extent of the statin effects on irisin. No *a priori* power calculations were made for changes in irisin since the primary outcome parameter of the study in the parent trial was change in LDL cholesterol. In univariate analyses some statistically significant correlations were found but alpha inflation cannot be excluded as the reason driving these associations. Especially stepwise linear regression procedures have been criticized to be prone for generating chance findings due to multiple testing. Therefore we abstained from further multivariate analysis model building. Lastly, the clinical relevance of our findings remains to be established.

Considering that irisin administration has been proposed as a potential therapeutic tool to treat obesity and diabetes [50] and thus may have implications for decreasing cardiovascular risk, our results are of potential clinical relevance.

*In conclusion,* our results show that simvastatin increases circulating irisin concentrations *in vivo* after two weeks of treatment in healthy individuals and also in HSKMCs *in vitro*. In HSKMCs, simvastatin-induced cellular stress markers as well as protective response markers, and inhibition of mitochondrial oxidative stress blocked simvastatin-induced irisin secretion, suggesting a role of oxidative stress in irisin regulation in human muscle. Whether the increase in irisin levels is a specific and metabolically important effect or whether it is related unspecifically to myocyte damage and/or cellular stress needs to be investigated further. If irisin eventually proves to be related to muscle damage, it might be speculated that irisin as circulating biomarker of muscle damage is much more sensitive than currently available markers. The results presented herein are suggestive of interconnections between the irisin and lipid metabolism pathways and warrant further study.
